# Liquid‒liquid phase separation: a potentially fundamental mechanism of sepsis

**DOI:** 10.1038/s41420-025-02599-2

**Published:** 2025-07-07

**Authors:** Huiyi Chen, Shunyi Huang, Longcheng Quan, Caiyuan Yu, Yang Zhu, Xiaocong Sun, Yuanli Zhang, Liehua Deng, Feng Chen

**Affiliations:** 1 Department of Critical Care Medicine, The Affiliated Hospital of Guangdong Medical University, 524000, Zhanjiang, China; 2 Yuebei People’s Hospital, The Affiliated Hospital of Shantou University Medical College, 512000, Shaoguan, China; 3 Department of Gerontology, The Affiliated Hospital of Guangdong Medical University, 524000, Zhanjiang, China

**Keywords:** Sepsis, Molecular biology

## Abstract

Sepsis is a life-threatening condition characterized by overactivated inflammation and a dysregulated immune response caused by infection. The predominant mechanism underlying the vulnerability and severity of sepsis has not been fully elucidated. Liquid‒liquid phase separation (LLPS) is a recently discovered, powerful mechanism that drives the formation of membraneless organelles and their biological functions. In particular, emerging evidence indicates that multiple core proteins involved in immune responses, inflammatory signalling, and programmed cell death are organized as protein condensates through LLPS. Here, we present an up-to-date review of the hypothesis that LLPS may underlie the fundamental mechanisms of sepsis, with a focus on the immune system response, changes in inflammatory signalling, and programmed cell death, with the goal of advancing our understanding of the pathological mechanisms of sepsis.

## FACTS

- Sepsis is one of the leading causes of morbidity and mortality in society, with an estimated 50 million cases and 11 million sepsis-related deaths worldwide each year.
- Sepsis is characterised by overactivated inflammation and a dysregulated immune response caused by infection.
- Multiple core proteins involved in immune responses, inflammatory signalling, and programmed cell death are organised as protein condensates through the mechanism of liquid‒liquid phase separation.
- An in-depth understanding of the mechanisms of LLPS in the immune response, inflammatory signalling, and programmed cell death will allow the discovery of new mechanisms and therapeutic targets for sepsis.

## OPEN QUESTIONS

- What are the mechanisms of LLPS in immune responses, inflammatory signalling, and programmed cell death?
- How does LLPS participate in the pathogenesis of sepsis?
- Is LLPS an important pathogenic mechanism of sepsis?

## Introduction

Sepsis is a well-characterized pathological syndrome associated with multiorgan dysfunction that is caused by sustained overactivated inflammation and host immune suppression after severe infection [1]. Sepsis remains a major cause of morbidity and mortality in society, with an estimated 50 million cases and 11 million sepsis-related deaths worldwide each year [2]. Sepsis is considered an unbalanced immune response in which pathogens invade protective defence mechanisms and multiply rapidly to directly and continuously damage host cells, leading to the exhaustion of immune cells and an imbalance in homoeostasis [3]. Therefore, exploring new detailed mechanisms related to immune signalling and the inflammatory response during sepsis is warranted for advancing sepsis treatment.

**Fig. 1:**
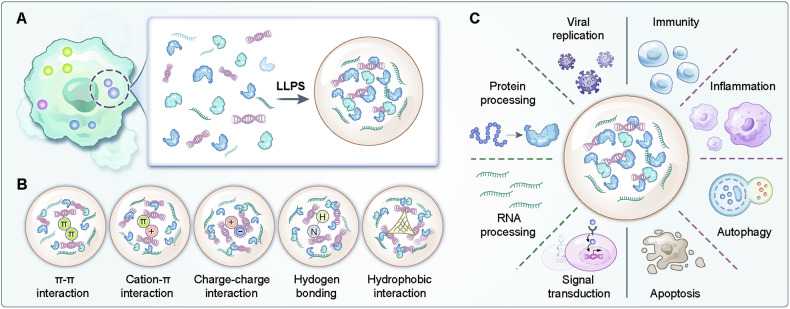
Formation mechanisms and core functions of LLPS. **A** LLPS, a fundamental biophysical phenomenon where biomolecules in solution separate into distinct fluid-like condensates, enables cellular regionalisation through the formation of membraneless organelles in eukaryotic cells. **B** IDRs drive phase separation via various weak interactions, including charge‒charge interactions, cation‒π interactions, π‒π stacking, hydrogen bonding, and hydrophobic interactions. **C** LLPS is involved in multiple biological processes, such as viral replication, signal transduction, RNA and protein processing, immune responses, inflammatory signalling, and programmed cell death.

Liquid–liquid phase separation (LLPS) is a vital and common phenomenon that forms the basis of membraneless organelles in eukaryotic cells and provides a novel principle for explaining the precise spatial and temporal regulation of living cells [4]. LLPS is driven mainly by multivalent interactions between macromolecules, which are strongly associated with specialised protein domains—the intrinsically disordered region (IDR) and low-complexity regions (LCRs) [5], involving electrostatic complementarity between cationic and aromatic residues manifesting as cation‒π bonding, π‒electron cloud stacking among aromatic amino acid side chains (π‒π interactions), charge‒charge pairing of oppositely polarised residues (ionic interactions), and the spatial alignment of polarised molecular dipoles [6] (Fig. 1). LLPS has the ability to induce dynamic and rapid interactions in biomolecular condensates within a cell, and this ability allows the cell to respond rapidly and adapt to changes in the external environment, thus playing a key role in various biological activities, such as the stress-induced DNA damage response and repair, granule assembly, genome organisation, metabolic regulation, neuronal synaptic signalling and signal transduction [7–9]. Recently, LLPS has emerged as one of the central principles used to explain multiple tumour-related activities [10, 11] and neurodegenerative biology [12, 13] but is a relatively new concept with respect to diseases in the field of critical illnesses, including sepsis. Notably, LLPS has been referred to as an important mechanism for the activation of the immune response, where the innate immune system provides the initial response to prevent infection [14, 15]. In addition, multiple core proteins or protein complexes involved in inflammatory signalling [16] and programmed cell death [17, 18] are organised as protein condensates through LLPS. These data suggest that phase separation may be an important mechanism involved in the pathogenesis of sepsis.

This review presents an integrated framework of the effects of LLPS on basic alterations associated with sepsis, including the immune system response, changes in inflammatory signalling, and programmed cell death, and discusses the mechanistic details, hoping to expand our understanding of the pathological mechanisms of sepsis and identify promising strategies for future therapeutics.

## Llps and the sepsis-related innate immune response

### LLPS drives NLRP6 inflammasome activation

**Fig. 2:**
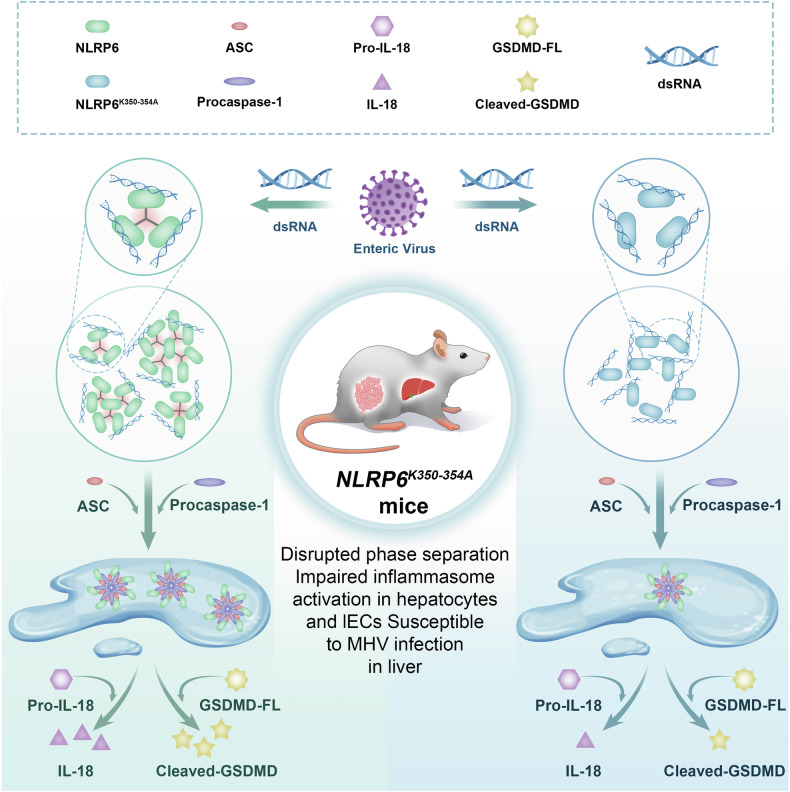
LLPS is involved in virus-induced activation of the NLRP6 inflammasome. NLRP6 interacts with dsRNA produced by various viruses to form liquid-like dsRNA‒NLRP6 droplets through LLPS. Droplets of dsRNA–NLRP6 formed through phase separation could serve as supramolecular organisation centres to recruit downstream molecules, such as apoptosis-associated speck-like protein containing CARD (ASC). ASC further recruits and activates caspase-1, with the subsequent initiation of the caspase-mediated processing of pro-IL-18 and the pore-forming protein gasdermin D (GSDMD) to promote the maturation and release of cytokines. A polylysine sequence (K350–354A) mutation abolishes inflammasome activation of NLRP6 by significantly weakening the dsRNA-induced LLPS of NLRP6, thus impairing antimicrobial defences.

Nucleotide-binding and oligomerization domain (NOD)-like receptors (NLRs) are a canonical type of pattern recognition receptor (PRR) that serve as the major component of the inflammasome, a high-molecular-weight complex that functions as an immune sensor of infection or cellular damage [19]. NLRP6 is a relatively newly identified member of the NLR family and acts as an inflammasome implicated in granulopoiesis, neutrophil recruitment and the formation of neutrophil extracellular traps (NETs) in bacterium-induced sepsis [20]. In addition, NLRP6 participates in initiating multiple pathways, including the inflammasome pathway and type I interferon pathway, and dampens nuclear factor-κB (NF-κB) and mitogen-activated protein kinase (MAPK) responses [21, 22]. A recent study revealed that NLRP6 regulates the antiviral immune response through LLPS-induced inflammasome activation [23] (Fig. 2). In that study, the authors provide biochemical and cellular evidence that double-stranded (ds) RNA, which is produced by various viruses, can bind directly to the C-terminal LRR domain of the inflammasome protein NLRP6 to form a liquid-like condensate. At high concentrations, NLRP6 can independently fulfil LLPS, while at low concentrations, it can form droplets upon interacting with dsRNA, indicating that LLPS may be an inherent property of NLRP6 and is not strictly dependent on dsRNA and that dsRNA has the ability to enhance the LLPS of NLRP6. NLRP6-dsRNA condensates function as a supramolecular organisation centre for recruiting downstream factors, and apoptosis-associated speck-like protein containing CARD (ASC) independently drives LLPS to form ASC droplets, which then fuse with the dsRNA-NLRP6 droplets, leading to enhanced dynamics of the droplets for the assembly of the inflammasome complex. The authors further revealed that the polylysine sequence (K350–354), an IDR region in the NACHT domain of NLRP6, plays a vital role in multivalent interactions and LLPS between NLRP6 and dsRNA. Specifically, the K350–354 A mutation did not affect NLRP6 expression but instead disrupted NLRP6 interactions and droplet formation, leading to decreased expression of interferon-stimulated genes, reduced IL-8 release and hyporesponsive inflammation. In addition, an NLRP6 ligand—lipoteichoic acid—facilitates NLRP6 LLPS, and a helicase involved in NLRP6-induced interferon signalling—DHX15—also fused with NLRP6-dsRNA condensates. In this context, LLPS of NLRP6 may represent a common response to ligand stimulation.

As a critical mediator of inflammasome formation, the NLRP3 inflammasome has several components in common with NLRP6, such as ASC and caspase-1 [24]. Some evidence has shown that LLPS may be present in the upstream regulatory system of NLRP3. For example, as a collagen-binding receptor tyrosine kinase, activated discoidin domain receptor 1 (DDR1) triggers an inflammatory response by activating the NLRP3 inflammasome [25]. A recent study reported that DDR1 facilitates yes-associated protein (YAP) signalling activation through LLPS, a process that depends on its transmembrane domain [26]. YAP specifically promotes NLRP3 inflammasome activation by physically interacting with NLRP3 and blocking its polyubiquitination [27]. Notably, YAP undergoes LLPS independently and recruits the transcriptional coactivators TAZ and TEAD4 into condensates to realise its assembly by LLPS as well [28, 29]. Additionally, galectin-3, a multifunctional carbohydrate-binding lectin, modulates inflammatory responses through the activation of the NLRP3 inflammasome [30, 31]. The unstructured N-terminal domain (NTD) of galectin-3 undergoes LLPS driven by interactions among its aromatic residues [30]. Notably, a very recently published paper reported that the signal-specific induction of NLRP3 LLPS serves as a molecular switch for its activation [32]. Key mechanistic insights include the following: ZDHHC7-mediated palmitoylation is essential for NLRP3 phase separation, which drives the formation of dynamic condensates with liquid-like properties; diverse activators—including potassium efflux inhibitors, the TLR7 agonist imiquimod, metabolic palmitate, and mitochondrial cardiolipin—converge on this unified pathway to trigger NLRP3 assembly; and structural analyses identify an IDR within the FISNA domain, where three evolutionarily conserved hydrophobic residues mediate multivalent weak interactions crucial for phase separation. Strikingly, site-specific mutations at these residues completely abrogate inflammasome activation. This study establishes a causal relationship between phase separation dynamics and inflammasome signalling, proposing the targeted modulation of the phase separation interface as a potential therapeutic paradigm for the treatment of NLRP3-driven pathologies such as sepsis.

Further studies are needed to determine whether phase separation is a broad organisation principle shared with other inflammasomes and to explore the biological mechanism of inflammasome phase separation and its role in inflammation and immune processes.

### LLPS is involved in TLR4-induced inflammation

**Fig. 3:**
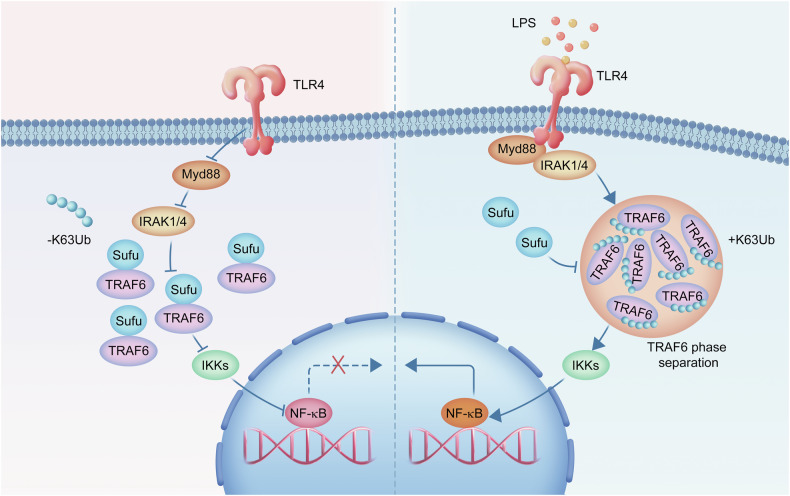
Sufu prevents sepsis by disrupting the LLPS of TRAF6. Left panel: Physiologically, TRAF6 is dispersed in the cytoplasm and does not mediate inflammation by effectively activating the transcription of NF-κB signalling. Right panel: Upon the activation of LPS signalling, TRAF6 condenses through the mechanism of LLPS, a previously undefined phenomenon. In addition, Sufu, an important member of the Hh signalling pathway, negatively regulates TLR-related inflammatory damage by disrupting the LLPS and droplet formation of TRAF6 upon LPS signalling. TRAF6 autoubiquitination and oligomerization are required for TRAF6 LLPS.

Toll-like receptors (TLRs) are a subfamily of PRRs implicated in the innate immune system that regulate the secretion of proinflammatory cytokines and promote innate and adaptive immune responses [33]. Tumour necrosis factor receptor-associated factor 6 (TRAF6) functions downstream of TLR4-induced inflammatory pathways as a primary adaptor, leading to multiorgan failure in individuals with sepsis [34]. A recent study revealed that TRAF6 undergoes LLPS during lipopolysaccharide (LPS) signalling and further revealed that Suppressor of Fused (Sufu), a key negative regulator of the Hedgehog signalling pathway, prevents LPS-induced NF-κB activation in sepsis by disrupting the LLPS and droplet formation of TRAF6 [16] (Fig. 3). Recently, TRAF6 LLPS has been identified as a novel antimicrobial immune regulatory mechanism against tuberculosis [35]. This process is driven by arginine residues within its helical domain and activates both the NF-κB and AP-1 signalling pathways in response to *Mycobacterium tuberculosis* (Mtb) infection. Moreover, TRAF6 is also a ubiquitin E3 ligase that promotes IKKγ LLPS and polyUb chain formation, leading to the subsequent activation of IκB kinase (IKK) complexes [36]. Upon stimulation by proinflammatory factors, IKKγ, a regulatory subunit of the IKK complex, drives LLPS via multivalent interactions with polyUb [37]. The induction of IKKγ LLPS depends on the ubiquitin-binding domain and zinc finger of IKKγ and is also required for NF-κB translocation into the nucleus. Interestingly, NLR family CARD domain containing-3 (NLRC3) negatively modulates TLR4-dependent signalling by directly binding to TRAF6, increasing its degradation and inhibiting its autoubiquitination [38]. These findings indicate that TRAF6 serves as a shared factor of NLR and TLR signal transduction and that LLPS-related TRAF6 activation may play a vital role in the crosstalk of different types of PRR activation.

ALPK1, a recently identified cytosolic pattern recognition receptor, recognises ADP-β-D-manno-heptose (ADP-Hep), a bacterial LPS metabolite, to activate the NF-κB signalling pathway [39]. In innate immune signalling, the TIFA protein forms oligomers following ALPK1 activation to facilitate downstream signalling, although the precise regulatory mechanism remains unclear. Recent research by Prof. Yin’s team revealed that ADP-Hep recognition by ALPK1 triggers TIFA activation and induces the co-phase separation of TIFA with TRAF6. This phase-separated compartment enables TRAF6 to exert its E3 ubiquitin ligase activity, catalysing K63-linked ubiquitin chain formation, which recruits downstream effectors to rapidly initiate inflammatory signalling [40]. This study elucidates a novel regulatory mechanism of the ALPK1-TIFA-TRAF6 axis and provides new strategic insights for the development of therapies targeting this pathway.

In summary, these data establish a direct link between phase segregation and sepsis-induced inflammation and suggest that phase separation may serve as a potential therapeutic target for sepsis-associated disorders.

### Phase separation participates in cGAS-related immunity

Cyclic GMP–AMP synthase (cGAS) is an important type of PRR that senses both viral DNA and mislocalized cellular DNA, such as mtDNA and cytoplasmic chromatin fragments [41]. Following the recognition of pathogenic or endogenous DNA, the cGAS enzyme is activated and catalyses the conversion of GTP and ATP to the second messenger 2’,3’-cyclic GMP–AMP (cGAMP), which binds to the adaptor protein STING to trigger immune and inflammatory responses [42, 43]. The cGAS-STING signalling cascade is highly involved in septic pathology [44]. Although profound progress has been made in understanding the regulatory mechanisms of cGAS activity in previous studies, how to strictly control cGAS activity to maintain immune homoeostasis is still unclear.

**Fig. 4:**
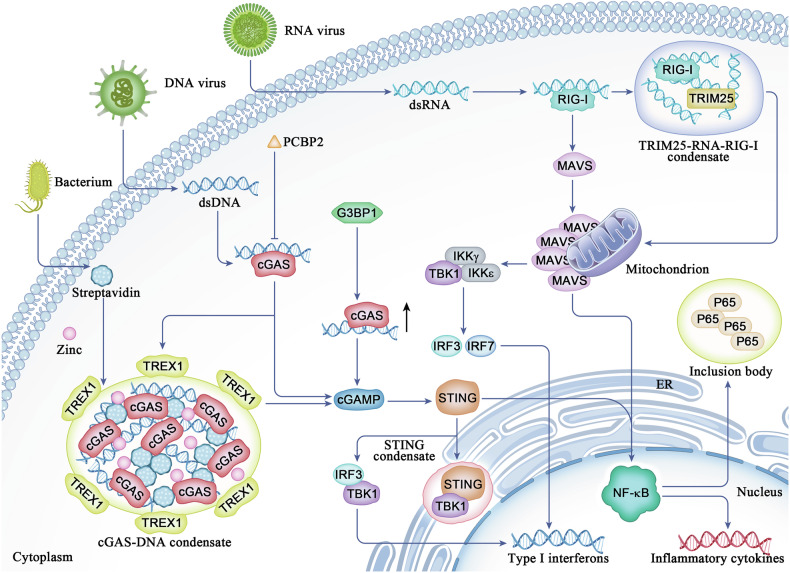
Phase separation is associated with cGAS-related inflammation. Following the recognition of dsDNA, cGAS forms liquid-like condensates to catalyse the synthesis of cGAMP, which in turn interacts with and activates the adaptor protein STING to trigger type I interferon production and inflammatory responses through TBK1 and IRF3 signalling. The LLPS of cGAS–DNA droplets not only inhibits TREX1 exonuclease activity but also restricts TREX1 to the droplet periphery. Specifically, the LLPS of cGAS–DNA prevents the TREX1-induced degradation of DNA to allow efficient sensing of pathogenic DNA, thus maintaining appropriate innate immune activation. Multiple cellular and viral proteins modulate the condensation of cGAS. For example, PCBP2 negatively regulates the cGAS-associated innate immune response to dsDNA virus infection by reducing the enzyme activity of cGAS. In contrast to PCBP2, G3BP1 promotes the phase separation of cGAS and cGAS-STING signalling by binding to cGAS. In addition, streptavidin, a pathogenic protein secreted from *Streptomyces avidinii*, promotes the phase separation of the cGAS‒DNA complex by interacting with cGAS. dsRNA drives the co-condensation of TRIM25 and G3BP1 to form stress granules, which are critical in activating the RNA sensor retinoic acid-inducible gene I protein (RIG-I) signalling pathway, thus restraining RNA virus infection. TRIM25 regulates RIG-I ubiquitination, promotes the phosphorylation of IRF7 and IRF3, and subsequently activates IFN-related immune responses.

A recent study indicated that the binding of dsDNA to cGAS robustly induced the formation of liquid-like cGAS‒DNA condensates in vitro and in vivo and that the N-terminal IDR of cGAS is responsible for condensate formation by increasing the valency of DNA binding [45] (Fig. 4). Functionally, the dsDNA-induced LLPS of cGAS promotes its enzymatic activity to increase cGAMP production and, in turn, activates STING signalling through IRF3 and TBK1, which play important roles in antiviral immunity. LLPS of cGAS–DNA not only promotes cGAS activity by preventing DNA degradation by the exonuclease TREX1 to allow the efficient sensing of cytosolic DNA but also restricts TREX1 to the droplet periphery, thus maintaining appropriate innate immune activation [46]. The activation of cGAMP leads to phase separation of STING on the endoplasmic reticulum (ER), which recruits TBK1 but excludes IRF3 to form the cGAS-STING-TBK1 axis, thereby preventing excessive activation of innate immunity [47]. IRF3 is activated not only by TBK1-triggered phosphorylation but also by SIRT1-mediated deacetylation. The deacetylation of IRF3 drives LLPS in response to viral infection, thereby forming a liquid condensate with a DNA-targeted IFN-stimulated response element, contributing to the upregulation of IFN-I and NLRP3 expression [48, 49].

In addition to immunostimulatory DNA, cGAS also forms phase separation condensates with cytoplasmic dsRNA, although RNAs do not promote the synthesis of cGAMP by cGAS [50, 51]. Notably, these findings are in line with previous structural data showing that cytosolic DNA rather than RNA sensing result in a conformational change that activates and synthases cGAS [52]. Thus, LLPS is insufficient for the activation of cGAS in the absence of the correct conformational change induced by DNA. However, studies have also revealed that RNAs stimulate the LLPS of cGAS and increase cGAS‒DNA condensation and cGAS enzymatic activity, especially in the presence of low DNA concentrations. However, whether the LLPS of cGAS shares a widely conserved process with other nucleic acid sensors remains an important research area. Another vital question that remains unanswered is whether the phase separation of cGAS–DNA is necessary for cGAS enzymatic activity. The above evidence highlights that the phase separation of cGAS is a required step for robust activation of the cGAS enzyme and synthesis of cGAMP. Recently, several studies have reported inconsistent results. One study reported that the phase separation of cGAS–DNA is dispensable for the overall level of cGAMP synthesis and does not directly govern cGAS enzyme activation, as specific cGAS mutants (K187N/L195R) have been shown to exhibit a more than 3-fold increase in cGAMP synthesis but not cGAS–DNA condensate formation [53]. These results indicated that the LLPS of cGAS–DNA and cGAS enzymatic activity are functionally distinct properties. Further research is needed to elucidate the exact relationship between phase separation and cGAS enzymatic activity, which is important for balancing pathogen recognition and cGAS-STING activation. In addition, the LLPS of cGAS–DNA is regulated by PCBP2, which specifically interacts with cGAS and then disrupts cGAS–DNA condensation, leading to the inhibition of cGAS enzyme activity and low activity of the cGAS-STING pathway [54]. In contrast to PCBP2, G3BP1 promotes the phase separation of cGAS and cGAS-STING signalling by interacting with cGAS. Moreover, cGAS activity is modulated by multiple factors, such as cellular and viral proteins and posttranslational modifications. For example, bacterial proteins can regulate cGAS LLPS. Streptavidin, a secreted bacterial protein produced by *Streptomyces*, binds to cGAS to promote cGAS‒DNA interactions, thereby promoting the LLPS of the cGAS‒DNA complex and facilitating cGAS activation [55]. Whether these factors regulate the enzyme activity of cGAS by affecting its phase separation and whether phase separation represents a common mechanism responsible for cGAS ligand stimulation need to be further investigated.

Notably, the serum lactate level has been recognised as an important biomarker for the diagnosis of sepsis, and hyperlactatemia represents the strongest indicator of sepsis outcomes. Moreover, monitoring the serum lactate level can guide the therapeutic response [56, 57]. A far more recent study revealed that the uptake of lactate dampens innate immunity through the mechanism of cGAS lactylation, which abrogates cGAS LLPS with DNA and enzyme activity [58]. However, the underlying mechanism by which hyperlactatemia leads to sepsis remains largely unknown, and whether the phase separation of cGAS plays a key role in this process is worth exploring. In addition, the stress granule protein DDX3X, which is involved in various sepsis-related pathological processes, such as infection, immunity and cell death [59], undergoes phase separation in a manner dependent on both its IDR and RNA-binding domain [60]. This study further demonstrated that DDX3X increases the condensation and activation of cGAS through its own phase separation ability. This evidence highlights the correlation between cGAS LLPS and sepsis, but this issue needs to be further explored. A deeper understanding of the mechanism of cGAS phase separation and the use of phase separation to trigger appropriate immune responses to pathogens may have beneficial effects on sepsis-related immunity.

### LLPS is involved in damage-associated molecular patterns (DAMPs)-induced inflammation

High mobility group box-1 protein (HMGB1) is a highly conserved nonhistone nuclear protein that has also been identified as a canonical DAMP that can be recognised mainly by TLRs and induces NF-κB translocation into the nucleus [61]. HMGB1 expression is substantially increased during polymicrobial sepsis, which is associated with a poor prognosis [62]. Recently, Denes et al. reported that mispartitioning into the nucleolus is highly involved in abnormal HMGB1 LLPS [63]. Specifically, HMGB1 undergoes LLPS via the IDR in the C-terminus. When a frameshift mutation replaces the IDR acidic tail of HMGB1 with an arginine-rich basic tail, HMGB1 with a mutant tail undergoes LLPS to form droplets at a lower concentration, attenuates the dynamic and liquid fluidity of HMGB1 droplets, and facilitates HMGB1 partitioning into nuclear condensates, culminating in mispartitioning into the nucleolus. However, a far more recent study revealed that HMGB1 can hardly undergo phase separation alone; HMGB1 can interact with the G-quadruplex of the KRAS promoter (GQ^KRAS^) and shows LLPS properties with GQ^KRAS^. HMGB1 facilitates and stabilises the GQ folding of GQ^KRAS^ by promoting the formation of the GQ^KRAS^–HMGB1 complex, which is fundamental for HMGB1 phase separation [64]. The reason for this difference may be that HMGB1 phase separation depends on its protein concentration. In sum, in addition to PRRs, DAMPs also exhibit LLPS properties, suggesting that LLPS may play a significant role in initiating inflammation and deserves further investigation with respect to its role during sepsis.

## Llps is involved in aberrant response of immune cells in sepsis

### Innate immune cells in sepsis

Septic patients and model mice exhibit elevated NET levels, which contribute to hyperinflammation [65, 66]. NETs are webs composed of decondensing chromatin, chemicals, cytokines and other functional molecules that are produced after neutrophil NETosis [67]. Recent studies have investigated the role of LLPS in the establishment of chromatin activity patterns [68].

Although few studies have reported the association between chromatin decondensation and LLPS, the involvement of LLPS in chromatin condensation has been widely reported. LLPS is identified as the primary phase separation in the nucleus, and multivalent LLPS interactions occur among chromatin-binding factors for chromatin condensation [69]. Nucleosomes are membraneless organelles, and their generation is recognised to be somewhat dependent on LLPS [70]. Single-stranded nucleic acids promote the formation of linker histone H1 condensates [71]. Multiple bromodomain proteins, such as BRD4 and H1, facilitate chromatin formation via LLPS and promote its compaction [69, 72]. Moreover, the nonhistone chromosome binding protein HP1 is highly involved in nuclear organisation and chromosome segregation [73]. The HP1α homologue undergoes LLPS to form droplets with dsDNA, core nucleosomes and aurora B kinase [74]. The charge of IDR-H is a crucial factor responsible for driving the LLPS of HP1 homologues. Unlike HP1α and HP1α, which carry basic IDR-H or IDR-N domains, HP1β, which has an acidic IDR, fails to form a liquid condensate on its own but undergoes LLPS with the help of histone H3K9me3. Future studies that investigate the association between LLPS and chromatin decondensation may be intriguing. With respect to NET-related signalling, NETs activate STING signalling upon TLR2 activation [75, 76] and promote the recognition of mtDNA by cGAS and the formation of cGAS–DNA condensates, leading to the induction of STING-TBK1-IRF3 signalling [77, 78]. STING condensates inhibit the subsequent activation of TBK1, IRF3 and NF-κB [47].

In summary, LLPS has been shown to play a role in NET generation, cGAS-STING signalling regulation in neutrophils, and macrophage activation in innate immunity.

### Adaptive immune cells in sepsis

**Fig. 5:**
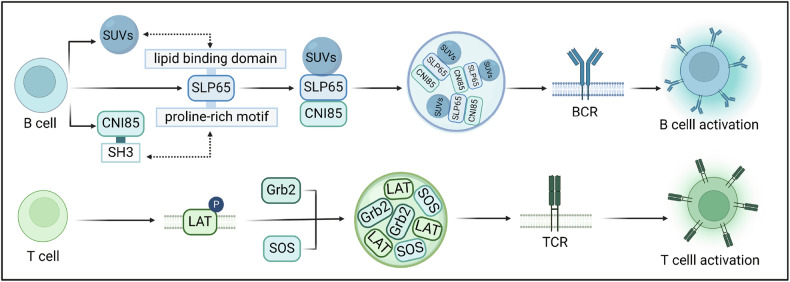
LLPS regulates immune cell alterations during adaptive immunity. SLP65 drives tripartite LLPS, forms droplets with CIN85 and SUVs, and activates BCR signalling pathways. Tripartite LLPS requires the interaction between the CIN85 SH3 domain and SLP65 proline-rich motifs and the preference of the lipid-binding domain for high-membrane-curvature SUVs. LAT phosphorylation drives LLPS and forms macromolecule condensates with Grb2 and SOS to trigger TCR signalling pathways. This figure was created with BioRender.com.

B-cell antigen receptor (BCR) signalling regulation requires the scaffold protein SH2 domain-containing leucocyte protein of 65 kDa (SLP65) [79]. SLP65 drives tripartite LLPS and forms droplets with Cbl-interacting protein of 85 kDa (CIN85) and small unilamellar vesicles (SUVs), culminating in efficient BCR signalling activation [79] (Fig. 5). Since the N-terminal lipid-binding domain of SLP65 is sensitive to membrane curvature, SLP65 favours anchoring to high-membrane-curvature SUVs [79]. SUVs promote SLP65 recruitment and increase SLPR6 enrichment in droplets [79]. The interaction between SLP6 and CIN85 is mediated by the SH3 domain of CIN85 and the proline-rich motif of SLP65 [79]. Together, the tripartite LLPS of SLP65–CIN85–SUVs is mediated by specific anchoring domains, SUVs at physiological concentrations and multivalent interactions between SLP65 and CIN85 [79]. Furthermore, T-cell receptor (TCR) signalling in T cells is activated by a transmembrane protein, i.e., linker for the activation of T cells (LAT). Tyrosine phosphorylation of LAT drives LLPS and results in macromolecule condensation with Grb2 and SOS under isothermal conditions [80–82]. In summary, with respect to adaptive immunity, LLPS may regulate BCR signalling activation via SLP65, DDR1, and NLRP6 and control TCR signalling via LAT.

## Llps is involved in programmed cell death in sepsis

During sepsis progression, uncontrolled cell death greatly contributes to the exhaustion of immune cells, followed by immunosuppression [83]. Here, the types of programmed cell death in sepsis, including autophagy, apoptosis, ferroptosis and pyroptosis, are discussed from the perspective of phase separation.

### Autophagy in sepsis

After stimulation, the mTORC1-mediated inhibition of TFEB is attenuated, and TFEB is activated [84]. TFEB undergoes LLPS through its bHLH domain and forms a liquid condensate with a low fusion propensity, high interfacial tension and rigid interfacial boundaries [85, 86]. TFEB LLPS is vulnerable to external conditions; for example, inositol polyphosphate multikinase and the small-molecule compound Ro-3306 both disrupt condensate stability and block TFEB-LLPS-related autophagy [85]. BRD4 also forms condensates with P300 downstream of TFEB signalling and inhibits the expression of autophagy-related genes [87]. RB1CC1/FIP200 is a crucial component of the autophagic induction complex, contains an IDR and undergoes LLPS independent of p62 and other autophagy-related proteins [88]. RB1CC1/FIP200 LLPS and its IDR acetylation are both required for autophagic modulation [88]. p62 contains folded recognition domains and structurally disordered binding motifs, which display properties of LLPS [89]. The phosphorylation of p62 at S403 promotes p62 binding to K63 polyubiquitin chains through the interaction between UBA and polyubiquitin chains, driving LLPS in a ubiquitin-valence-dependent manner and the formation of p62 bodies with liquid-like properties [90]. Additionally, specific E3 ubiquitin protein ligase 1 (Smurf1) not only facilitates the formation of p62 droplets via LLPS [91] but also mediates the exchange of molecules with the p62 droplet periphery and ameliorates Nrf2 activation in a p62-LLPS-dependent manner; in turn, Nrf2 is required for Smurf1, NBR1 and p62 expression [91]. Thus, a positive feedback loop of p62 LLPS forms, and the loop is blocked by Smurf1-mediated degradation of the p62 condensate—a type of LLPS intervention [91]. The p62-binding protein DAXX drives p62 LLPS and regulates p62 recruitment [92]. For degeneration, mTORC1 mediates PGL-1/-3 phosphorylation and promotes PGL-1/-3 to undergo LLPS, resulting in the generation of PGL granules, which exhibit anti-autophagy degradation properties [93]. In addition, PTK6 (protein tyrosine kinase 6), a nonreceptor tyrosine kinase, promotes LLPS of HNRNPH1 by phosphorylating it at the Y210 site [94]. This LLPS facilitates the HNRNPH1-mediated retention of exon 10 in NBR1, activating selective autophagy. In nonselective autophagy, the Atg1 complex undergoes LLPS to form condensates anchored to vacuolar membranes via Atg13‒Vac8 interactions, establishing phagophore assembly sites (PASs) for autophagosome initiation [95]. For selective autophagy, cargo-specific receptors or phase-separated complexes (e.g., Atg11 foci) orchestrate autophagosome formation [96]. Atg11 foci, critical early hubs in selective autophagy, recruit machinery proteins (Atg1 and Atg9) through low-affinity ligand‒receptor interactions that sustain receptor mobility, enabling the dynamic assembly of initiation centres. Conversely, high-affinity interactions impair Atg11 focus formation and inhibit autophagy. Notably, Atg11 focus assembly occurs independently of downstream factors (Atg9 and Vac8), highlighting its role as an LLPS-driven early platform for the recruitment of the autophagy machinery, which is distinct from later maturation steps.

### Apoptosis in sepsis

LLPS is involved in the stability of the p53-mediated transcriptional initiation of apoptosis [97]. p53 undergoes LLPS, induces the transcription of apoptotic genes and induces BAX/BAK-caspase-3 signalling [97]. p53 forms liquid condensates in 45 mM NaCl at slightly acidic pH values, indicating the environmental dependence of LLPS [98]. The LLPS of p53 depends on its disordered unstructured basic region, the integrity of its tetramerization domain [99], the multivalent electrostatic interactions of its NTD and CTD, and the distances between the core domains and the CTDs [100]. Moreover, p53 LLPS requires a specific interaction between specific DNA and p53 and p53 Ser392 phosphorylation to promote droplet dynamics and increase the p53 content in the condensate with increasing numbers and sizes [101]. ssDNA, dsDNA, and ATP function as downstream regulators of p53 LLPS [100]. Peptides designed through molecular dynamics simulations have high affinity for the N‑terminal IDR of p53 and subsequently ameliorate p53 LLPS [98], indicating that the design of peptides is a novel direction for the LLPS-targeted treatment of sepsis. p53 promotes cGAS-STING-IRF3-mediated apoptosis [102], and IRF3 and BAX form a dynamic protein complex with Tom70 and Hsp90 to mediate virus-induced apoptosis [103]. The LLPS of STING and IFR3 also affects their signalling activity [47, 49]. SHP1 (also known as Ptpn6) is a cytoplasmic phosphatase that regulates caspase-8-mediated apoptosis during sepsis [104, 105]. SHP1 is able to undergo LLPS, a process that is associated with the SHP1-R360E mutation [106]. Moreover, the homologous protein SHP2 regulates apoptosis [107]. SHP2 is an allosteric enzyme that undergoes LLPS via the PTP domain with multivalent interactions [108]. The formation of SHP2 LLPS requires the open conformation of SHP2; LLPS is inhibited when SHP2 maintains a closed conformation, for example, upon binding a SHP2 allosteric inhibitor [108]. SHP2 is involved in the STING-IFR3 axis and regulates STING-IRF3-induced apoptosis [48, 109], which may be associated with STING and IFR3 LLPS [49].

### Other types of cell death in sepsis

Ferroptosis is a newly identified form of cell death triggered by lethal iron-dependent lipid peroxidation [110]. Ferroptosis suppressor protein 1 (FSP1) is a ferroptosis inhibitor that prevents lipid peroxidation independent of glutathione [18]. A specific FSP1 inhibitor facilitates the formation of FSP1 condensates in cells and in a cell-free system, along with hyperactive ferroptosis [18]. Specifically, the FSP1 LLPS property requires the presence of an IDR and LCRs, N-terminal myristoylation and different amino acid residues. In addition, ferroptosis induced by the long noncoding RNA lncFASA is regulated by the LLPS of peroxiredoxin 1 [111]. BRD4 reportedly controls genome activity via LLPS [77], and BRD4 upregulates FSP1 expression and prevents ferroptosis [112]. STING-related innate immunity is associated with ferroptosis [113], and the cGAS-STING-IFR3 axis is also regulated by LLPS [47, 49]. These results collectively indicate that LLPS may have indispensable functions in the dysregulation of ferroptosis during sepsis progression.

Pyroptosis is a specific form of cell death triggered by the gasdermin family of proteins, which have pore-forming abilities, and uncontrolled pyroptosis is related to a wide-range of organ impairments during sepsis [114, 115]. Both NLRP6 and NLRP3 participate in pyroptosis regulation [114, 116], and the LLPS properties of NLPR6 and NLRP3 upstream regulators have been described above [117]. HMGB1 mediates pyroptosis via the TLR4-NF-κB-NLRP3 axis [118], and the formation of HMGB1-LLPS influences protein function [63, 119]. Galectin-3 promotes caspase-4/11-mediated pyroptosis in macrophages during sepsis and drives LLPS via its NTD interaction [117, 120]. STING and IRF3 undergo LLPS, and their signalling also regulates pyroptosis. These findings suggest that the LLPS of NLRP6, HMGB1, galectin-3, STING and IFR3 is a potential target for the pyroptosis-directed treatment of sepsis.

Together, LLPS plays extensive roles in various types of sepsis-related cell death. Given the complicated effects of LLPS, further studies are needed to explore its potential clinical application in sepsis.

## Conclusions and perspectives

Here, we systematically summarise the emerging correlations and mechanisms between LLPS and the pathological processes associated with sepsis. Although a variety of key proteins associated with sepsis can undergo LLPS, which is involved in the fundamental functions of these proteins, only a few studies have explored whether the LLPS of these proteins is present under sepsis conditions and influences septic pathogenesis. Therefore, more studies are needed to identify other sepsis-associated proteins with LLPS properties and, more importantly, to elucidate the precise mechanisms underlying their effects on sepsis. One important protein that should be mentioned is pattern recognition receptor triggering receptor expressed on myeloid cells-1 (TREM-1), which is expressed on immune cells and is characterised as a major player in the pathophysiology of sepsis by amplifying the immune response and inflammatory cascades, especially through synergism with TLR signalling [121]. Currently, TREM-1 is considered a promising target for the clinical treatment of sepsis [122, 123]. Notably, the activation of TREM-1 does not depend on its expression level on the cell membrane but rather on its membrane polymerisation, which controls signal transduction, and TREM-1 is further stabilised by its endogenous ligands and the adaptor molecule DAP12 [124]. However, the molecular mechanisms by which TREM-1 clusters and dimerises at monocyte and neutrophil membranes under inflammatory conditions are unclear. Interestingly, phase separation is an important mechanism for maintaining the formation of protein aggregates, and TREM-1 harbours a wide range of IDRs, which are widely known as critical structures for the formation of diverse protein liquid droplets, supporting the theoretical possibility that the aggregation of TREM-1 on the cell membrane is mediated by phase separation. If this assumption is validated, it will provide new insights into the mechanisms of the TREM-1-mediated amplification of inflammation and immune signalling and even reveal a new approach for sepsis treatment.

Importantly, the biological changes discussed above essentially occur during sepsis. Sepsis is a complicated systemic disease triggered by diverse factors, and different organs have different specificities. For example, recent studies established that LLPS represents an important organisation principle of synaptic formation and function [125, 126]. In addition, increasing evidence supports the hypothesis that the phase separation of pathological proteins may be a triggering factor for protein aggregation in neurodegenerative disorders [127, 128]. These synaptic and cognitive-associated proteins or protein complexes, such as Tau and amyloidβ, are also important risk factors for sepsis-associated encephalopathy (SAE) [129, 130]. Whether phase separation, which is involved in neuronal synaptic signalling, plays an important role in the pathology of SAE deserves further exploration.

In this study, we elucidate the pivotal role of phase separation in modulating immune responses, inflammatory signalling, and programmed cell death. Crucially, these processes are not isolated phenomena but constitute an integrated molecular network in the pathogenesis of sepsis. Therefore, investigating the role of phase separation in sepsis should focus on its cooperative mechanisms across these processes, as targeting such interdependencies may yield multipronged therapeutic benefits. In conclusion, phase separation provides promising mechanisms for understanding sepsis-related regulation of immunity, the inflammatory response and the programmed cell death process, suggesting that LLPS may be a fundamental mechanism of sepsis. Future studies should expand from in vitro experiments to animal models and include more physiological conditions postmortem to better elucidate the role of LLPS in the pathogenesis of sepsis. Rapid advances in computational simulations and nuclear magnetic resonance imaging technologies are anticipated to benefit the modulation of LLPS, thus providing promising therapeutic strategies for the treatment of sepsis.
